# NY-ESO-1 tumour associated antigen is a cytoplasmic protein detectable by specific monoclonal antibodies in cell lines and clinical specimens

**DOI:** 10.1054/bjoc.2000.1251

**Published:** 2000-06-15

**Authors:** E Schultz-Thater, C Noppen, F Gudat, U Dürmüller, P Zajac, T Kocher, M Heberer, G C Spagnoli

**Affiliations:** Research Division, Department of Surgery, University of Basel, ZLF, 20 Hebelstrasse, Basel, 4031, Switzerland; Institute of Pathology, University of Basel, 40 Schönbeinstrasse, Basel, 4003, Switzerland

**Keywords:** tumour associated antigens, cancer testis antigens, immunodetection, immunotherapy

## Abstract

NY-ESO-1 gene encodes a novel member of the cancer/testis (CT) family of human tumour-associated antigens (TAA). Specific monoclonal antibodies (mAb) have identified the corresponding gene product in lysates of tumour cell lines as a 22 kDa protein but no data are available concerning its intracellular location or distribution within neoplastic tissues. We have generated NY-ESO-1 specific mAbs recognizing the target molecule in cytospin preparations and in sections from clinical tumour specimens. These reagents identify NY-ESO-1 TAA in melanoma cell lines expressing the specific gene as a cytoplasmic protein, sharing the intracellular location of most MAGE TAA. In a series of 12 melanoma specimens, specific staining, limited to neoplastic cells, was detectable in the five cases where NY-ESO-1 gene expression was observed. In two of them over 90% of tumour cells showed evidence of positive staining. Lower percentages of positive neoplastic cells ranging between single cells and 50% were observed in the remaining tumours. These data suggest that active specific immunotherapies targeting NY-ESO-1, alone or in combination with other TAA could be of high clinical relevance in sizeable subgroups of melanoma patients. © 2000 Cancer Research Campaign


*British Journal of Cancer* (2000) **83**(2), 204–208
© 2000 Cancer Research Campaign
doi: 10.1054/ bjoc.2000.1251, available online at http://www.idealibrary.com on IDEAL®

### NY-ESO-1 tumour associated antigen is a cytoplasmic protein detectable by specific monoclonal antibodies in cell lines and clinical specimens

**E Schultz-Thater[1,3], C Noppen[1,3], F Gudat[2], U Dürmüller[2], P Zajac[1], T Kocher[1], M Heberer[1] and GC Spagnoli[1]**

[1]Research Division, Department of Surgery, University of Basel, ZLF, 20, Hebelstrasse, 4031, Basel, Switzerland [2]Institute of Pathology, University of Basel, 40, Schönbeinstrasse, 4003, Basel, Switzerland [3]The first two authors equally contributed to this work.

**Summary** NY-ESO-1 gene encodes a novel member of the cancer/testis (CT) family of human tumour-associated antigens (TAA). Specific monoclonal antibodies (mAb) have identified the corresponding gene product in lysates of tumour cell lines as a 22 kDa protein but no data are available concerning its intracellular location or distribution within neoplastic tissues. We have generated NY-ESO-1 specific mAbs recognizing the target molecule in cytospin preparations and in sections from clinical tumour specimens. These reagents identify NY-ESO-1 TAA in melanoma cell lines expressing the specific gene as a cytoplasmic protein, sharing the intracellular location of most MAGE TAA. In a series of 12 melanoma specimens, specific staining, limited to neoplastic cells, was detectable in the five cases where NY-ESO-1 gene expression was observed. In two of them over 90% of tumour cells showed evidence of positive staining. Lower percentages of positive neoplastic cells ranging between single cells and 50% were observed in the remaining tumours. These data suggest that active specific immunotherapies targeting NY-ESO-1, alone or in combination with other TAA could be of high clinical relevance in sizeable subgroups of melanoma patients. © 2000 Cancer Research Campaign

**Keywords**: tumour associated antigens; cancer testis antigens; immunodetection; immunotherapy

Tumour-associated antigens (TAA) of the cancer testis (CT) family are characterized by a peculiar expression pattern since the corresponding genes are transcribed in healthy testis and in a number of histologically unrelated tumours (Boon and van der Bruggen, 1996; Chen et al, 1998). In particular, epitopes from different MAGE TAA have been shown to be recognized by specific cytotoxic T cells (CTL), and, more recently, to induce class II restricted T cell responses (Boon and van der Bruggen, 1996; Chaux et al, 1999; Manici et al, 1999). Clinical trials based on the use of these antigens have been initiated and promising preliminary results have been reported (Marchand et al, 1995; Marchand et al, 1999; Nestle et al, 1999; Thurner et al, 1999).

Recently, novel members of the CT TAA family have been identified by taking advantage of a newly devised technique, SEREX (Sahin et al, 1995; Türeci et al, 1998), that relies on the recognition of target molecules by specific antibodies present in patients' sera. One of them, NY-ESO-1, emerges as a potential candidate for the inclusion in therapeutic vaccine preparations since it encompasses both humoral and CTL target epitopes (Chen et al, 1997; Jäger et al, 1998; Wang et al 1998).

We and others have previously generated monoclonal antibodies (mAbs) specific for MAGE family CT TAA (Chen et al, 1994; Schultz-Thater et al, 1994; Kocher et al, 1995; Carrel et al, 1996; Jurk et al, 1998). By demonstrating the widespread expression of target determinants in defined tumour types, these reagents support the implementation of specific immunization procedures (Gudat et al, 1996; Hofbauer et al, 1997). So far, NY-ESO-1 specific mAb identifying this TAA in 'western blot' assays have been reported (Jäger et al, 1998), but no reagents capable of recognizing it in cytological or histological preparations have been described.

We report here on the generation and characterization of mAbs recognizing NY-ESO-1 TAA, permitting its identification as a cytoplasmic protein detectable in discrete cell lines and clinical tumour samples.

*Received 21 December 1999*
*Revised 9 March 2000*
*Accepted 15 March 2000*

*Correspondence to: GC Spagnoli*

### MATERIALS AND METHODS

### Cell lines

D10, MZ-2 and A375 cell lines are gifts of Dr Rimoldi (Ludwig Institute, Lausanne, Switzerland). HBL and S7 cell lines were kindly provided by Drs Ghanem (Free University of Brussels, Belgium) and Shibahara (Tohoku University, Sendai, Japan), respectively. RE cell line is a gift of Dr Siegrist (University of Basel, Switzerland), whereas WM-266 cell line was obtained from American Type Culture Collection (Rockville, MD). SK-MEL-37 cell line is a gift of Dr Jungbluth (Ludwig Institute at Memorial Sloan Kettering Cancer Center, New York, NY).

### Preparation of NY-ESO-1 fusion protein

NY-ESO-1 entire gene (courtesy of Dr Sahin, Homburg/Saar, Germany) was cloned into pET-32a vector (Novagen, Madison, WI) allowing inducible expression of inserted genes in the form of fusion proteins containing thioredoxin and a six histidin tail.

The plasmid was used to transform BL21(pLysS) *E. coli* strain. After a three hour induction in the presence of IPTG (1 mM final concentration) bacterial cultures were spun down and recombinant proteins were purified under native conditions upon binding to nickel resins (Ni-NTA, Qiagen, Basel, Switzerland) and elution in the presence of 250 mM imidazole. Production and purification of the recombinant proteins were monitored by SDS-PAGE and Coomassie blue staining (Schultz-Thater et al, 1994).

### Production of monoclonal antibodies

BALB/c mice were repeatedly injected i.p. at two week intervals with 100 μg Ni purified material containing NY-ESO-1 gene product. The first immunization included complete Freund's adjuvant, whereas the second and third were performed in the presence of incomplete Freund's adjuvant. Three days after a last injection in the absence of adjuvant preparations, animals were sacrificed and fusions were carried out as previously detailed (Schultz-Thater et al, 1994). Screening of HAT resistant hybridoma supernatants was performed by ELISA.

### Detection of NY-ESO-1 gene expression

Total cellular RNA was extracted from the cell lines under investigation (see above), reverse transcribed and tested in 25 cycles RT-PCR assays in the presence of primer pairs specific for β-actin positive control gene yielding a 661 bp amplicon (Schultz-Thater et al, 1994) or NY-ESO-1 gene yielding a 354 bp amplicon (Chen et al, 1997). Each cycle included a 30 s denaturation step at 94°C, a 40 s annealing step at 72°C and a 40 s extension at 72°C. RT-PCR products were run on 1.5% agarose gels in the presence of ethidium bromide and photographs were taken under UV transillumination.

### Immunocytochemistry and immunohistochemistry

Acetone fixed cytospin preparations of the indicated melanoma cell lines were incubated in the presence of undiluted control or specific hybridoma supernatants for 30 min at room temperature. Frozen tumour sections were thawed, washed in PBS, fixed in paraformaldehyde-lysine-periodate (Gudat et al, 1996) or buffered formalin and incubated overnight at 4°C in the presence of hybridoma supernatants. For both preparations, bound antibodies were visualized by using the APAAP method according to the recommendations of the supplier (DAKOPATTS A/S, Glostrup, Denmark).

### RESULTS

### Production of recombinant NY-ESO-1

NY-ESO-1 entire open reading frame was subcloned into the pET 32a inducible expression vector. Upon IPTG treatment, the TAA could be successfully produced in soluble form in the context of a fusion protein, inclusive of thioredoxin and a poly-histidin tail (data not shown). Following purification on nickel columns, the fusion protein comprising NY-ESO-1 TAA was detectable in Coomassie blue stained gels with an apparent molecular weight of 46 kDa. This material was used to immunize mice and screen hybridomas. Similarly produced soluble thioredoxin served as negative control in screening procedures.

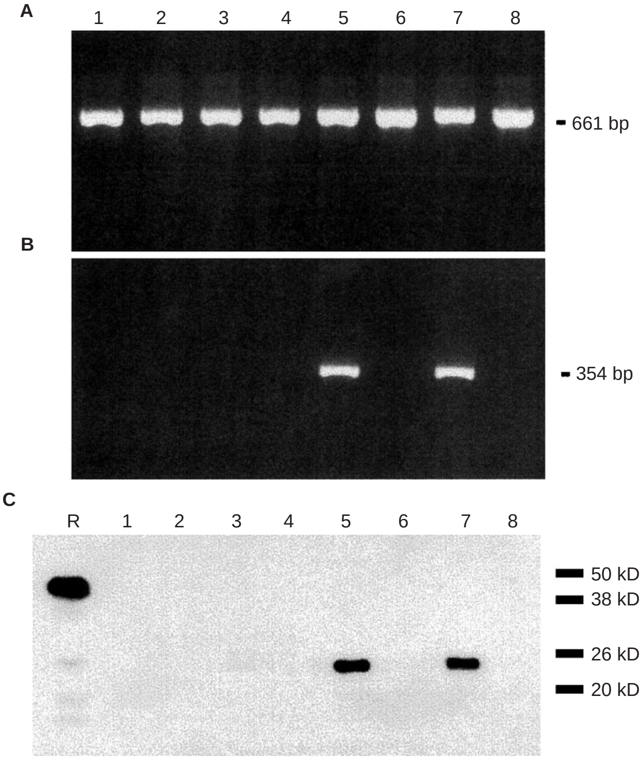

**Figure 1** Immunochemical detection of NY-ESO-1 gene product in melanoma cell lines. Total cellular RNA was extracted from D10, HBL, MZ-2, S7, SK-MEL-37, A375, RE and WM-266 cell lines (lanes 1 to 8, respectively), reverse transcribed and amplified in 25 cycles RT-PCR in the presence of β-actin specific (panel A) or NY-ESO-1 (panel B) specific primers. Amplification products were run in the presence of ethidium bromide on agarose gels that were then photographed under UV transillumination. Lysates from the same cell lines (lanes 1 to 8, respectively) and the recombinant fusion protein containing NY-ESO-1 gene product (lane R) were run on PAGE under reducing conditions. Gels were then blotted and assayed in the presence of B9.8 hybridoma supernatant

### Generation of NY-ESO-1 specific mAbs recognizing recombinant and native gene products

Five mAbs (B4.2, IgG2a; B9.8, IgG1; B10.3, IgG1, D8.10, IgG1 and D8.38, IgG1) appeared to recognize specifically recombinant NY-ESO-1 protein in ELISA assays. In order to assess the capacity of the mAbs produced to identify the native protein, eight melanoma cell lines were characterized in terms of expression of NY-ESO-1 gene by 25 cycles RT-PCR. All lines expressed β-actin house-keeping gene, whereas NY-ESO-1 gene expression was only detectable in positive control SK-Mel 37 (Jäger et al, 1998) and in RE cell lines (Figure 1, panels A and B). Lysates from all cell lines were then tested together with the recombinant fusion protein in immunoblot assays in the presence of the mAbs under investigation. All reagents recognized the positive control recombinant fusion protein (Figure 1, panel C, lane R). Most importantly, they identified a single band of an apparent molecular weight of 24 kDa, in lysates from both SK-MEL-37 and RE cell lines, expressing NY-ESO-1 gene, as detectable in RT-PCR assays. Representative data obtained by using B9.8 mAb are shown in Figure 1, panel C. In contrast, no reactivity could be observed against lysates derived from D10, HBL, MZ-2, S7, A375 or WM266 melanoma cell lines. Indeed, these lines failed to express NY-ESO-1 gene, although some of them expressed MAGE family TAA (Kocher et al, 1995).

*© 2000 Cancer Research Campaign* 

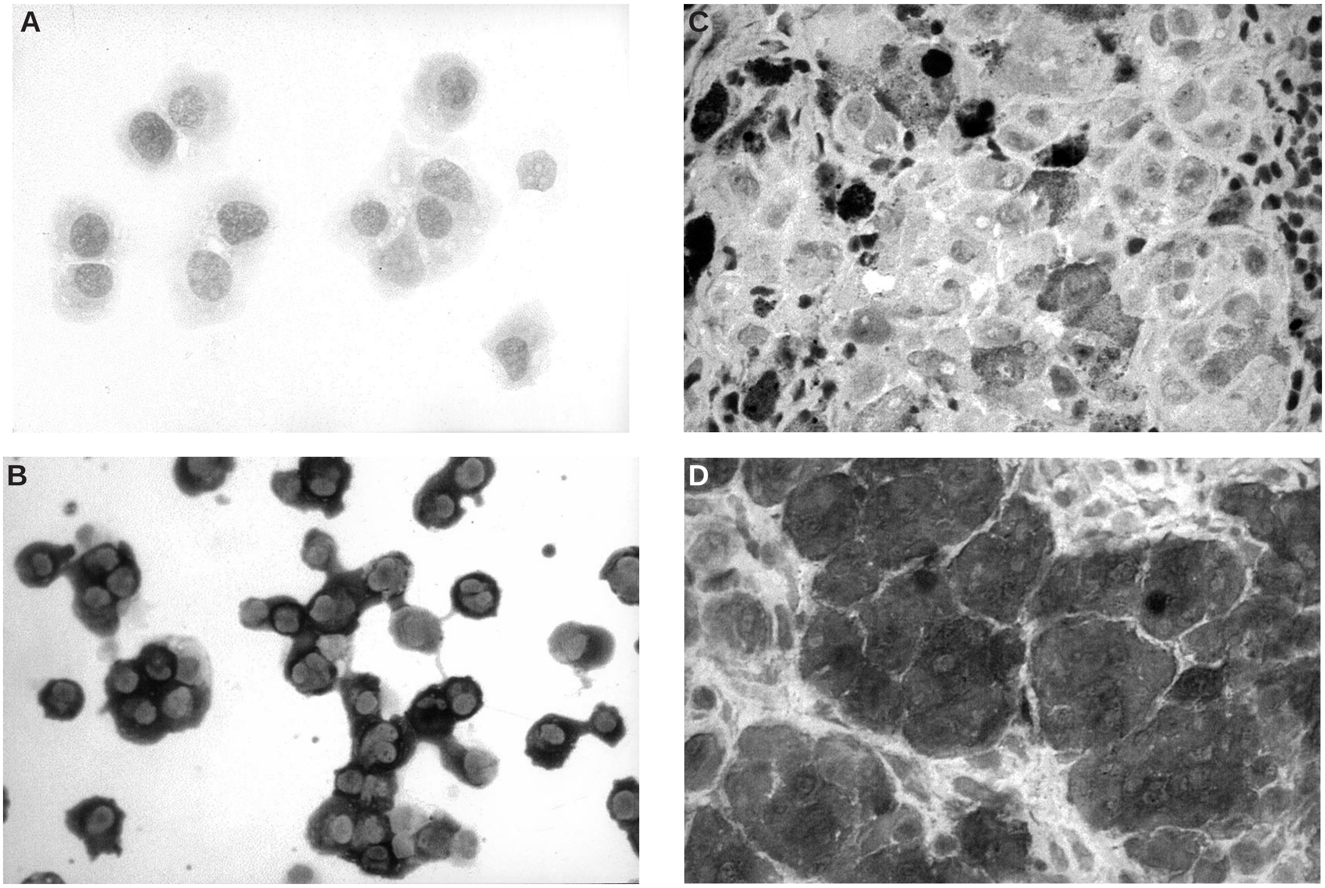

**Figure 2** Immunocytochemical and immunohistochemical detection of NY-ESO-1 gene product. Acetone fixed cytospin preparations of D10 or SK-MEL-37 (panels A and B) cells were incubated in the presence of B9.8 mAb. Fresh frozen sections from a nodular primary melanoma showing evidence of NY-ESO-1 gene expression (case no 9 from Table 1), were tested in the presence of control mAb (panel C) or B9.8 mAb (panel D). For all preparations specific mAb binding was revealed by APAAP technique. Melanin containing tumour cells are visible in panel C

### Immunocytochemical recognition of NY-ESO-1 TAA by specific mAbs

The identification of the target TAA in tumour cell lines was then attempted. Cytospin preparations of D10 or SK-MEL-37 cells were incubated in the presence of hybridoma supernatants, including appropriate isotype matched reagents and specific binding was revealed by APAAP method. Neither cell line was stained by irrelevant control mAb (data not shown). On the other hand, upon incubation with the mAbs under investigation, D10 cells scoring negative in PCR assays for NY-ESO-1 gene expression were not stained, whereas a strong cytoplasmic reactivity was indeed detectable in PCR positive SK-MEL-37 cells. Interestingly, in the latter cells no evidence of surface expression of the target antigen could be observed in flow-cytometry studies. Representative examples, obtained following staining of the two cell lines with B9.8 mAb are reported in Figure 2 (panels A and B).

### Detection of NY-ESO-1 gene product in clinical tumour samples

The possibility to detect NY-ESO-1 protein was then investigated in a series of 12 melanomas. Expression of NY-ESO-1 gene was first tested by 30 cycles RT-PCR. While positive control β-actin gene was found to be expressed in all samples, NY-ESO-1 transcripts were only amplified, to different extents, in five specimens (Figure 3, panel B). Fresh frozen sections were then studied in immunohistochemistry. Indeed, staining of tumour cells was observed in the specimens where evidence of specific gene transcription had been reported (Table 1). Percentages of cells showing expression of different intensity ranged between single cells and over 90% of neoplastic cells. Importantly, the staining, mostly detectable in the cell cytoplasm, appeared to be limited to cancer cells. Figure 2, panels C and D depicts sections from case no 9, as described in Table 1.

### DISCUSSION

NY-ESO-1 gene encodes a novel member of the CT TAA family (Chen et al, 1997; Jäger et al, 1998; Wang et al, 1998). Interestingly, NY-ESO-1 TAA has been shown to represent the molecular target of humoral immune responses in relatively high percentages of tumour patients (Stockert et al, 1998). Most importantly, HLA-A2 restricted epitopes recognized by CTL have been demonstrated (Jäger et al, 1998; Wang et al, 1998). NY-ESO-1 gene product was identified in immunoblots as a 22-kDa protein by a specific mAb (Jäger et al, 1998) but no data were reported concerning immunocytochemical and immunohistochemical detection or intracellular location.

 *© 2000 Cancer Research Campaign*

**Table 1** Detection of NY-ESO-1 gene product in melanoma

| Case no | Diagnosis | Localization | RT-PCR[a] | Positive cells[b] | Intensity[c] |
|---|---|---|---|---|---|
| 1 | Metastatic | Lymphnode | + | <50% | +/++ |
| 2 | Metastatic | Lymphnode | +/– | single cells | +/++ |
| 3 | Metastatic | Lymphnode | – | – | – |
| 4 | Metastatic | Lymphnode | – | – | – |
| 5 | Metastatic | Skin | – | – | – |
| 6 | Metastatic | Lymphnode | – | – | – |
| 7 | Metastatic | Lymphnode | – | – | – |
| 8 | Metastatic | Lymphnode | + | >90% | +/++ |
| 9 | Primary | – | + | >90% | +/+++ |
| 10 | Metastatic | Lymphnode | – | – | – |
| 11 | Metastatic | Skin | – | – | – |
| 12 | Metastatic | Skin | + | <50% | +/++ |

[a] RT-PCR data are reported in Figure 3, panel B; [b] Number of cells showing positive staining upon incubation with B9.8 mAb; [c] Intensity of reaction: + = weak, ++ = medium, +++ = strong

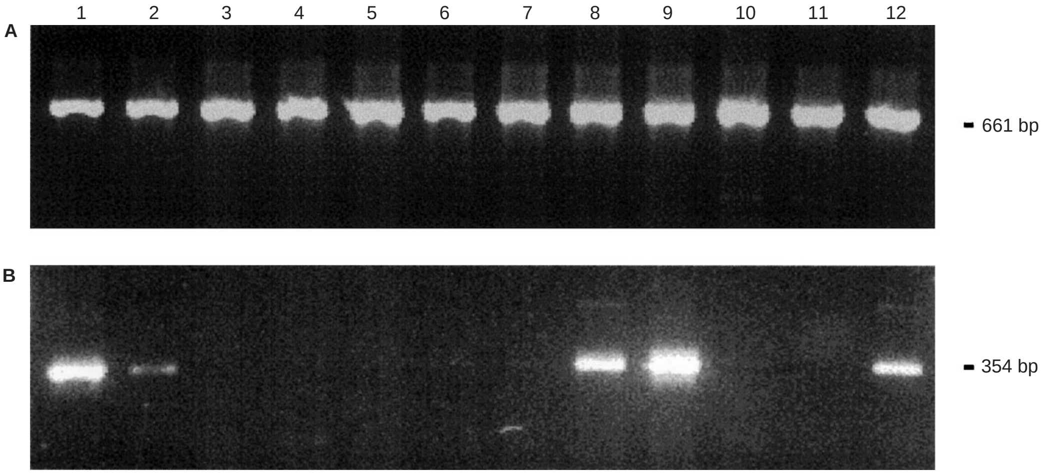

**Figure 3** Total cellular RNA was extracted from 12 different melanoma specimens (see Table 1), reverse transcribed and amplified in 30 cycles RT-PCR in the presence of β-actin specific (panel A) or NY-ESO-1 (panel B) specific primers. Amplification products were run in the presence of ethidium bromide on agarose gels that were then photographed under UV transillumination

We have produced NY-ESO-1 specific mAbs by using as immunogen a recombinant fusion protein including the TAA under investigation together with thioredoxin. These reagents recognize the native protein in lysates of melanoma cell lines expressing the specific gene in the apparent absence of cross-reactivities with other CT TAA such as MAGE family determinants. Most importantly, the NY-ESO-1 target molecule can be identified by these mAbs in immunocytological and immunohistological preparations. The specificity of this recognition is strongly supported by RT-PCR and immunoblot data, as related to the cell lines and tissues tested. Clearly, however, the possibility of cross reactivities within a family of highly homologous determinants (Lethé et al, 1998), similarly to MAGE TAA, cannot be formally ruled out (Van Baren et al, 1999).

Although sharing defined characteristics, NY-ESO-1 and MAGE TAA display specific features. At difference with MAGE, NY-ESO-1 gene is expressed in ovary tissue (Chen et al, 1997). Furthermore, NY-ESO-1 molecule appears to contain a C-terminal hydrophobic tail, potentially serving as membrane-associated domain (Chen et al, 1997), whereas MAGE family TAA do not. MAGE gene products are mostly characterized by a cytoplasmic location (Chen et al, 1994; Schultz-Thater et al, 1994; Kocher et al, 1995; Carrel et al, 1996). However, MAGE-11 protein has been shown to be prevailingly detectable in nuclei (Jurk et al, 1998).

Our data indicate that NY-ESO-1 gene product is detectable as a cytoplasmic protein in both cultured cell lines and clinical melanoma specimens, whereas no obvious evidence of specific staining of cell surfaces or nuclei was observed. Clearly, ultrastructural studies are required to clarify the role, if any, played by NY-ESO-1 potential transmembrane domain in intracellular location.

Staining with specific mAbs reveals intratumour heterogeneity in the expression of NY-ESO-1 TAA. Moreover, tumour cells displaying different staining intensities can be detected, closely resembling the MAGE TAA expression pattern (Gudat et al, 1996; Hofbauer et al, 1997). It is remarkable, however, that in a subgroup of the melanoma specimens tested in this work NY-ESO-1 gene product appears to be detectable in >90% of neoplastic cells. Although larger studies are obviously required, it is tempting to speculate that, in these patients, NY-ESO-1 derived epitopes could represent critical components of vaccine preparations.

The reagents described here together with the previously reported MAGE family specific mAbs could allow the evaluation of whether CT TAA expression is clustered in discrete cellular

*© 2000 Cancer Research Campaign* 

subsets or can be detected independently for discrete gene families. Furthermore, considering their relatively high specificity for neoplastic cells, they could be of use in complementing defined diagnostic and staging procedures.

### ACKNOWLEDGEMENTS

Thanks are due to Dr U Sahin (Homburg/Saar, Germany), to Dr U Certa (Basel, Switzerland) and to Dr A Jungbluth (New York, NY) for gifts of plasmids and cell lines, and to Dr G De Libero (Basel, Switzerland) for help in hybridoma technology. This work was partially supported by the Swiss Cancer League, the Regional Cancer Leagues of Basel-Stadt and Basel-Land, the San Salvatore (Lugano) and the Karl Mayer (Liechtenstein) Foundations and the Swiss National Fund for Scientific Research (grant no. 31–45560.95).

### REFERENCES

Boon T and van der Bruggen P (1996) Human tumor antigens recognized by T lymphocytes. *J Exp Med* **183**: 725–729

Carrel S, Schreyer M, Spagnoli G, Cerottini J-C and Rimoldi D (1996) Monoclonal antibodies against recombinant-MAGE-1 protein identify a cross-reacting 72-kDa antigen which is co-expressed with MAGE-1 protein in melanoma cells. *Int J Cancer* **67**: 417–422

Chaux P, Vantomme V, Stroobant V, Thielemans K, Corthals J, Luiten R, Eggermont AM, Boon T and van der Bruggen P (1999) Identification of MAGE-3 epitopes presented by HLA-DR molecules to CD4(+) T lymphocytes. *J Exp Med* **189**: 767–778

Chen Y-T, Stockert E, Chen Y, Garin-Chesa P, Rettig WJ, van der Bruggen P, Boon T and Old LJ (1994) Identification of the MAGE-1 gene product by monoclonal and polyclonal antibodies. *Proc Natl Acad Sci USA* **91**: 1004–1008

Chen Y-T, Scanlan MJ, Sahin U, Türeci O, Gure AO, Tsang S, Williamson B, Stockert E, Pfreundschuh M and Old LJ (1997) A testicular antigen aberrantly expressed in human cancers detected by autologous antibody screening. *Proc Natl Acad Sci USA* **94**: 1914–1918

Chen Y-T, Güre AO, Tsang S, Stockert E, Jäger E, Knuth A and Old LJ (1998) Identification of multiple cancer/testis antigens by allogenic antibody screening of a melanoma cell line library. *Proc Natl Acad Sci USA* **95**: 6919–6923

Gudat F, Zuber M, Dürmüller U, Kocher T, Schaefer C, Noppen C and Spagnoli GC (1996) The tumour-associated antigen MAGE-1 is detectable in formalin-fixed paraffin section of malignant melanoma. *Virchows Arch* **429**: 77–81

Hofbauer GFL, Schaefer C, Noppen C, Böni R, Kamarashev J, Nestle FO, Spagnoli GC and Dummer R (1997) MAGE-3 immunoreactivity in formalin-fixed, paraffin embedded primary and metastatic melanoma: frequency and distribution. *Am J Pathol* **151**: 1549–1553

Jäger E, Chen Y-T, Drijfhout JW, Karbach J, Ringhoffer M, Jäger D, Arand M, Wada H, Noguchi Y, Stockert E, Old LJ and Knuth A (1998) Simultaneous humoral and cellular immune response against cancer-testis antigen NY-ESO-1: definition of human Histocompatibility Leukocyte Antigen (HLA)-A2-binding epitopes. *J Exp Med* **187**: 265–270

Jurk M, Kremmer E, Schwarz U, Forster R and Winnacker EL (1998) MAGE-11 protein is highly conserved in higher organisms and located predominantly in the nucleus. *Int J Cancer* **75**: 762–766

Kocher T, Schultz-Thater E, Gudat F, Schaefer C, Casorati G, Juretic A, Willimann T, Harder F, Heberer M and Spagnoli GC (1995) Identification and intracellular location of MAGE-3 gene product. *Cancer Res* **55**: 2236–2239

Lethé B, Lucas S, Michaux L, De Smet C, Godelaine D, Serrano A, De Plaen E and Boon T (1998) LAGE-1, a new gene with tumor specificity. *Int J Cancer* **76**: 903–908

Manici S, Sturniolo T, Imro MA, Hammer J, Sinigaglia F, Noppen C, Spagnoli G, Mazzi B, Bellone M, Dellabona P and Protti MP (1999) Melanoma cells present a MAGE-3 epitope to CD4+ cytotoxic T cells in association with histocompatibility leukocyte antigen DR11. *J Exp Med* **189**: 871–876

Marchand M, Weynants P, Rankin E, Arienti F, Belli F, Parmiani G, Cascinelli N, Bourlond A, Vanwijck R, Humblet Y, Canon J-L, Laurent C, Naeyaert J-M, Plagne R, Deramaeker R, Knuth A, Jäger E, Brasseur F, Herman J, Coulie PG and Boon T (1995) Tumor-regression responses in melanoma patients treated with a peptide encoded by gene MAGE-3. *Int J Cancer* **63**: 883–885

Marchand M, van Baren N, Weynants P, Brichard V, Dreno B, Tessier M-H, Rankin E, Parmiani G, Arienti F, Humblet Y, Bourlond A, Vanwijck R, Liénard D, Beauduin M, Dietrich P-Y, Russo V, Kerger J, Masucci G, Jäger E, De Greve J, Atzpodien J, Brasseur F, Coulie PG, van der Bruggen P and Boon T (1999) Tumor regressions observed in patients with metastatic melanoma treated by an antigenic peptide encoded by gene MAGE-3 and presented by HLA-A1. *Int J Cancer* **80**: 219–230

Nestle FO, Alijagic S, Gilliet M, Sun Y, Grabbe S, Dummer R, Burg G and Schadendorf D (1999) Vaccination of melanoma patients with peptide- or tumor lysate-pulsed dendritic cells. *Nature Med* **4**: 328–332

Sahin U, Türeci O, Schmitt H, Cochlovius B, Johannes T, Schmits R, Stenner F, Luo G, Schobert I and Pfreundschuh M (1995) Human neoplasms elicit multiple specific immune responses in the autologous host. *Proc Natl Acad Sci USA* **92**: 11810–11813

Schultz-Thater E, Juretic A, Dellabona P, Lüscher U, Siegrist W, Harder F, Heberer M, Zuber M and Spagnoli GC (1994) MAGE-1 gene product is a cytoplasmic protein. *Int J Cancer* **59**: 435–439

Stockert E, Jäger E, Chen Y-T, Scanlan MJ, Gout I, Karbach J, Arand M, Knuth A and Old LJ (1998) A survey of the humoral immune response of cancer patients to a panel of human tumor antigens. *J Exp Med* **187**: 1349–1354

Thurner B, Haendle I, Röder C, Dieckmann D, Keikavoussi P, Jonuleit H, Bender A, Maczek C, Schreiner D, von den Driesch P, Bröcker EB, Steinman RM, Enk A, Kämpgen E and Schuler G (1999) Vaccination with Mage-3A1 peptide-pulsed mature monocyte-derived dendritic cells expands specific cytotoxic T cells and induces regression of some metastases in advanced stage IV melanoma. *J Exp Med* **190**: 1669–1678

Türeci O, Sahin U, Schobert I, Koslowski M, Schmitt H, Schild HJ, Stenner F, Seitz G, Rammensee HG and Pfreundschuh M (1998) The SSX-2 gene, which is involved in the t(X;18) translocation of synovial sarcomas, codes for the human tumor antigen HOM-MEL-40. *Cancer Res* **56**: 4766–4772

Van Baren N, Brasseur F, Godelaine D, Hames G, Ferrant A, Lehmann F, André M, Ravoet C, Doyen C, Spagnoli GC, Bakkus M, Thielemans K and Boon T (1999) Genes encoding tumor-specific antigens are expressed in human myeloma cells. *Blood* **94**: 1156–1164

Wang RF, Johnston SL, Zeng G, Topalian SL, Schwartzentruber DJ and Rosenberg SA (1998) A breast and melanoma-shared tumor antigen: T cell responses to antigenic peptides translated from different open reading frames. *J Immunol* **161**: 3598–3606

 *© 2000 Cancer Research Campaign*
